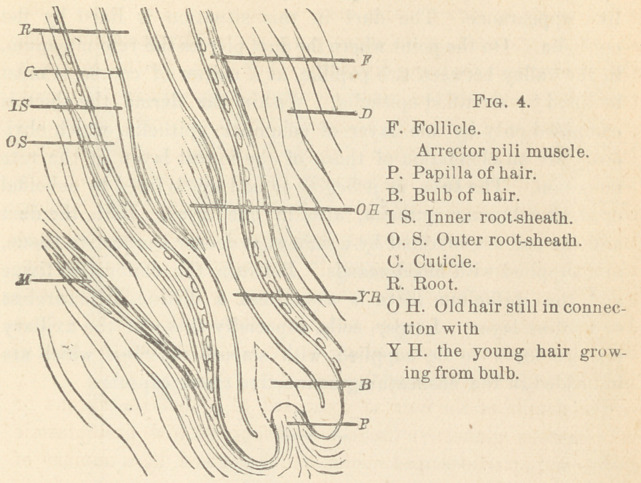# A Contribution to the Minute Anatomy of the Skin

**Published:** 1881-12

**Authors:** C. Heitzmann

**Affiliations:** New York


ARTICLE II.

A CONTRIBUTION TO THE MINUTE ANATOMY OF THE SKIN. BY C. HEITZMANN, M.D., of New York. Read before the American Dermatological Association, Newport, R. I., Sept. 1, 1881.

The apparently complicated structure of the integument becomes easily understood, if we keep in mind that there are but two main tissues entering the structure of the skin, viz., connective tissue and epithelium. The connective tissue produces the flat layer, called derma; the epithelium covers the derma on its outer surface. The boundary line between the two formations is not even, but fluted, supplied with numerous small protrusions of the derma, the so-called papillæ, the sum total of which bears the name "papillary layer." The bundles of the connective tissue everywhere run an oblique course; they are arranged in the shape of a coarse reticulum in the lowest portion of the derma, whose rhomboidal meshes contain a varying amount of fat-globules, the so-called subcutaneous tissue. In the derma proper, the bundles run in two almost rectangularly interlaced directions, thus producing a very dense felt, which by being tanned gives the leather. On the lowest portions of the derma the bundles are relatively coarse; they become the finer the nearer the papillary layer, and in the latter very delicate connective tissue fibers are noticeable only without a distinct arrangement into bundles. The epithelial formations on the top of the derma, again, exhibit two main layers, the lower one, that nearest to the papillary layer, being alive and supplied with nerves, the so-called rete mucosum; while the outermost layer is composed of dry, horny epithelia, giving the formation called epidermis.

The connective tissue is supplied with blood-vessels and lymphatics; the epithelium lacks such formations.

If we now imagine that the connective tissue, together with the covering epithelium, were a pliable sheet, for instance, of chamois, and we produce a depression of this sheet with one of our fingers—the result will be a pouch, whose innermost layers are epithelial, whose outermost layer is connective tissue. The epidermis will cover the inner surface of the pouch, and now bear the name "inner root-sheath;" next to this will be a layer, formed by the epithelia of the rete mucosum, which will be the "outer root-sheath;" the outside of the pouch must be connective tissue, and will represent the "follicle." On the bottom of the pouch will be a protrusion of the follicle, kindred to those on the surface of the skin, therefore connective tissue, the "papilla of the hair."

On our diagram slight alterations must be made. The epidermis, which is composed of a large number of flat epithelia, greatly varying in accordance with the width of this layer, upon entering the pouch and becoming the inner root-sheath, will gradually be reduced to a limited number of horny epithelia, in the midst of the pouch to not more than two strata. Near the bottom of the pouch the number of the epithelia again increases, the inner root-sheath gains in width, and is composed of three or four strata of epithelia, which have lost their horny character, and become of a protoplasmic nature again. The rete mucosum enters the pouch in its full width, but gradually becomes thinner, namely, composed of a smaller number of epithelia, which retain their original protoplasmic character, and at last, near the bottom of the pouch, after being reduced to a single layer, completely disappear.

Imagine, now, that against the bottom of the epithelial pouch, which, according to the main direction of the connective tissue bundles, runs in an oblique direction, a pin is pressed and the pouch turned upward again. This procedure, of course, will involve the inner root-sheath exclusively, and an elongation must result, of an epidermal character, according to the main character of the inner root-sheath. This elongation represents the hair.

The hair, therefore, is a solid elongation of the hollow inner root-sheath, and produced by the inner root-sheath alone. The outer root-sheath has nothing to do with the formation of the hair. On the bottom of the pouch there is a knob, composed of living protoplasmic epithelia, like those of the inner root-sheath in the same situation. This knob is called the bulb of the root of the hair, and directly surmounts the papilla of the hair. Higher up the epithelia become horny once more, and go to build up the root and the shaft of the hair.

Imagine, lastly, that on the side of the acute angle of the obliquely implanted pouch, the outer root-sheath, as said before, an off-shoot of the rete mucosum, be pushed laterally and downward by a pin, the result will be a third elongation, produced by the outer root-sheath, a small pouch itself, bearing the name "sebaceous gland." According to this diagram, the sebaceous gland is exclusively a formation of the outer root-sheath, while the inner root-sheath has nothing to do with the formation of the gland.

The idea of the production of the root-sheaths, the hair and the sebaceous glands, is illustrated by the accompanying diagrammatic figure 1. The explanation is as follows:

FIG. 1.

E. Epidermis.

R M. Rete mucosum.

P L. Papillary layer.

I S. Inner root-sheath.

O S. Outer root-sheath.

F. Follicle.

D. Derma.

M. Arrector pili muscle.

P. Papilla of the hair.

B. Bulb.

C. Cuticle.

R. Root.

S. Shaft of the hair.

S G. Sebaceous gland.

The epidermis, bulging downward, results in the formation of the inner root-sheath, while the rete mucosum, elongated downward, results in the formation of the outer root-sheath.

The bundles of the connective tissue of the derma, which give an outer investment to the pouch, composed of both root-sheaths, produce the follicle. Its innermost portion exhibits cross-sections of smooth muscle-fibers.

The papilla of the hair is a product of the follicle. Around the papilla is a knob, the bulb of the root of the hair, which continues into the root of the hair—that part inclosed in the pouch; and the shaft of the hair—that part standing forth on the surface of the skin.

The diagram shows that the inner root-sheath, upon approaching the bottom of the pouch, becomes widened, and on the bottom of the pouch turns over, thus first producing the bulb, afterward the root, and the shaft of the hair itself. The innermost layer of the inner root-sheath, by turning over, results in the formation of the cuticle, the single investing layer of both the root and the shaft of the hair.

The figure demonstrates, furthermore, that the outer root-sheath, upon approaching the bottom of the pouch, grows thinner and perishes at last, while on one side the outer root-sheath produces the pouch of the sebaceous gland. Between the outer root-sheath and the follicle there is a homogeneous layer, the so-called "structureless membrane." The arrector pili muscle is in connection with the muscle-layer of the follicle, and surrounds the bottom of the sebaceous gland.

The diagram serves as a key, which enables us to easily comprehend all formations of the skin engaged in the formation of the hair. First, let us study the upper portion of the hair-pouch from a specimen of the human skin, fig. 2. The pouch, as a rule, has a funnel-shaped widening on the surface of the skin, which is covered by stratified epidermal scales. These scales are traceable in direct union to the inner root-sheath, which begins on the so-called neck of the pouch, being composed of two epidermal layers only, and in honor of the discoverer, termed *Henle's* sheath. Next to the inner root-sheath lies the extremely delicate cuticle of the hair, which ensheaths both the root and the shaft of the hair. With higher powers we see on each hair finely serrated edges, the slightly bulging edges of the cuticle. The hair is composed of closely packed, horny epidermal spindles, which hold a varying amount of pigment granules. The rete mucosum directly elongates into the outer root-sheath, and this into the sebaceous gland. It is only the duct of this gland which is covered by flat horny epithelia, while the gland, as such, is

FIG. 2.

E. Epidermis.

R M. Rete mucosum.

P L. Papillary layer.

D. Derma.

F. Follicle.

M. Arrector pili muscle.

S G. Sebaceous gland.

O S. Outer root-sheath.

I S. Inner root-sheath.

C. Cuticle.

R. Root of hair.

Magnified 150 diam.

composed of cuboidal epithelia, like any acinous gland. The duct of the sebaceous gland, as a rule, empties into the funnel-shaped widening of the pouch, in the space between the inner root-sheath and the hair, or, more particularly, its covering cuticle. The outer root-sheath is composed of several strata of epithelia, like the rete mucosum itself. The strata are cuboidal epithelia, and it is only the stratum next to the derma, or, more particularly, the structureless membrane, which is formed by columnar epithelium. The inner surface of the structureless membrane, again, is covered by extremely delicate, flat endothelia, as first demonstrated, with the aid of silver-tinction, by *Czerny*. These endothelia, by very marked prickles and thorns, are directly connected with the adjacent columnar epithelia. To the presence of these thorns *D. Haight* first drew attention.

The pouch of the sebaceous gland is under the control of the arrector pili muscle, which represents a flat fan-like sheet, whose broad ends terminate in the papillary layer, while the narrow end is inserted in the follicle of the root of the hair. No doubt, the evacuation of the sebaceous gland is done by contraction of this muscle-sheet. The fatty mass will be squeezed first into the funnel of the hair-pouch, as a rule, and only on large sebaceous glands directly to the surface.

The lower extremity of the hair-pouch, in specimens taken from the human skin, is not readily comprehensible, unless upon the basis of the study of hairs of animals, especially of those strong hairs on the upper lip of kittens. This, perhaps, is the reason why, after many years' lively writing, not one author has given a plain description of the relations. As a matter of course, the essentials are identical in the hairs of kittens and those of man, though the former are, as a rule, more plain than the latter.

Fig. 3 illustrates the bottom of a hair-pouch from the lip of a kitten.

The inner root-sheath in its upper portion shows the light, horny *Henle's* layer. In an oblique line there appear polyhedral epithelia; at first pale and finely granular, with indistinct nuclei; deeper down coarsely granular and slightly elongated. This latter portion of the inner root-sheath represents what has been termed *Huxley's* layer. It is seen that on the bottom of the pouch this layer turns over, surrounds the papilla and constitutes the bulb of the root of the hair. The epithelia on the lower periphery of the papilla are columnar, gradually changing into the cuboïdal form, and more upward becoming elongated, spindle-shaped. Lastly, they emerge into the horny spindles which produce the main bulk of the hair. The boundary line between the inner root-sheath and the root of the hair is given by a thin, apparently structureless layer, outside of which is the inner root-sheath, inside the cuticle of the hair. The cuticle on the upper

FIG. 3.

F. Follicle.

T. Transverse sections of connective tissue bundles of derma.

M. Arrector pili muscle.

I S. Inner root sheath.

O S. Outer root-sheath.

P. Papilla.

C. Cuticle.

R. Root of hair.

H. Hyaline, or so called structureless membrane.

Magnified 300 dia portion of the root is composed, as well as on the shaft, of thin, imbricated scales, whose edges are slightly bulging forth over the surface of the hair, and give the latter the peculiar serrated appearance. Gradually the epithelia of the cuticle of the root assume a columnar shape and become nucleated. At the height of the bulb these columnar epithelia are very large, paler, granular, supplied with large and distinct nuclei. Their characteristic row runs in the middle between *Huxley's* layer and the bulb, at last blending with the cuboidal epithelia of both formations. Outside of the cuticular row there is another thin layer of flattened, pale epithelia, which evidently corresponds to the innermost structureless layer of the inner root-sheath. The middle portion of the bulb is often occupied by globular, indifferent or medullary corpuscles, which hold a varying amount of pigment, and fill also the central portion of the root, the so-called medullary space, which even in strong hairs may be absent. The outer root-sheath is composed, in its upper portions, of stratified epithelia, the outermost layer being distinctly columnar. The latter row is the last one left, as the outer root-sheath approaches the region of the bulb, and gradually becoming thinner, in turn is entirely lost at the height of the bulb, whose formation it does not enter at all. The boundary line between the outer and the inner root-sheath is again marked by the presence of a so called structureless membrane. Outside the outer root-sheath we find the follicle, a connective tissue formation, with interspersed circular muscle-spindles, in connection with those of the arrector pili muscle. Between the follicle and the outer root-sheath there is usually a broad homogeneous layer, which can be traced around the bulb of the root and the papilla of the hair.

The papilla of the hair is composed of a delicate fibrous or myxomatous connective tissue, freely supplied with protoplasmic bodies and spindle-shaped nuclei, and traversed by a number of capillary blood-vessels. The apex of the papilla in our specimen is not distinctly separated from the epithelia of the hair. The line of demarcation, however, as a rule, is distinguished by the presence of a row of columnar epithelia, or the medullary corpuscles.

Outside of the follicle there is the fibrous connective tissue of the derma, built up by longitudinal and transverse bundles.

In comparing what I have said about the theory of the formation of the hair with specimens of the skin, a satisfactory congruence will be found. This theory, as I have taught it for nearly seven years in my laboratory, will explain the fact that the inner root-sheath is withdrawn simultaneously with the root upon pulling a hair. It furthermore explains the process of shedding and the new formation of the hair.

We know through *Kölliker* and *C. Langer* that the young hair is formed around the old papilla. We know that at a certain height above the papilla there is a knob-like thickening (*Henle*), which corresponds to the bulb of the falling hair. The fact that

I add is that the new growth of a hair takes place within the realm of the inner root-sheath exclusively.

Fig. 4 is a diagram of the process of shedding. The inner

FIG. 4.

F. Follicle.

Arrector pili muscle.

P. Papilla of hair.

B. Bulb of hair.

I S. Inner root-sheath.

O. S. Outer root-sheath.

C. Cuticle.

R. Root.

O H. Old hair still in connection with

Y H. the young hair growing from bulb.

root-sheath, below the bulb of the old hair, which latter is fringed by the torn epidermal scales, gradually becomes widened. On the bottom of the pouch it turns over and produces the bulb, which is composed of medullary, or indifferent or embryonal corpuscles. The boundary between the two portions of the inner root-sheath is established by the cuticle, which, below the bulb of the old hair, is composed of columnar epithelia. The pigment, where there is any, lies exclusively in the central portion of the inner root-sheath, the future hair. The outer root-sheath has nothing to do with the new formation of the hair. The smooth muscles of the follicle evidently play a part in the process, inasmuch as through their contraction a narrow neck is established around the young hair, as first suggested by *Biesiadecki.*

With the views here advocated, also, the formation of the sudoriparous glands becomes understood. We simply may assume an elongation of the outer epithelial layers into the depth of the derma. The duct within the epidermal stratum shows windings, which, on places where the epidermis is thin, scarcely are perceptible, while on places, such as the soles of the feet, where the epidermis is very thick, they exhibit the characteristic corkscrew-like appearance. The duct in this situation is lined by flat epithelia. On the point where the duct pierces the rete mucosum, in the valley between two papillæ, as a matter of course, it must be lined by stratified epithelia. Within the derma the duct is composed only by one layer of columnar epithelia, which obviously are an elongation of those of the lowest layer of the rete mucosum. The tube, as it begins to coil up, is lined by cuboidal or short columnar epithelia, also in one layer. Both the duct and the coil are invested by a somewhat denser connective tissue, and supplied with blood-vessels. Between the connective tissue and the epithelium there is sometimes a distinct structureless or hyaline layer. Larger coils, especially those of the axillary pit, we know to be supplied with smooth muscles, which are imbedded in the ensheathing connective tissue capsule.

## Figures and Tables

**Fig. 1. f1:**
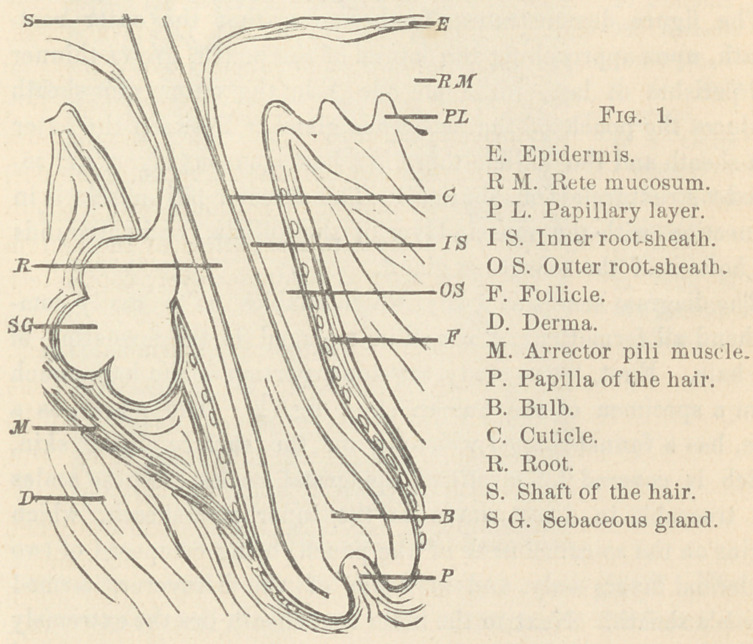


**Fig. 2. f2:**
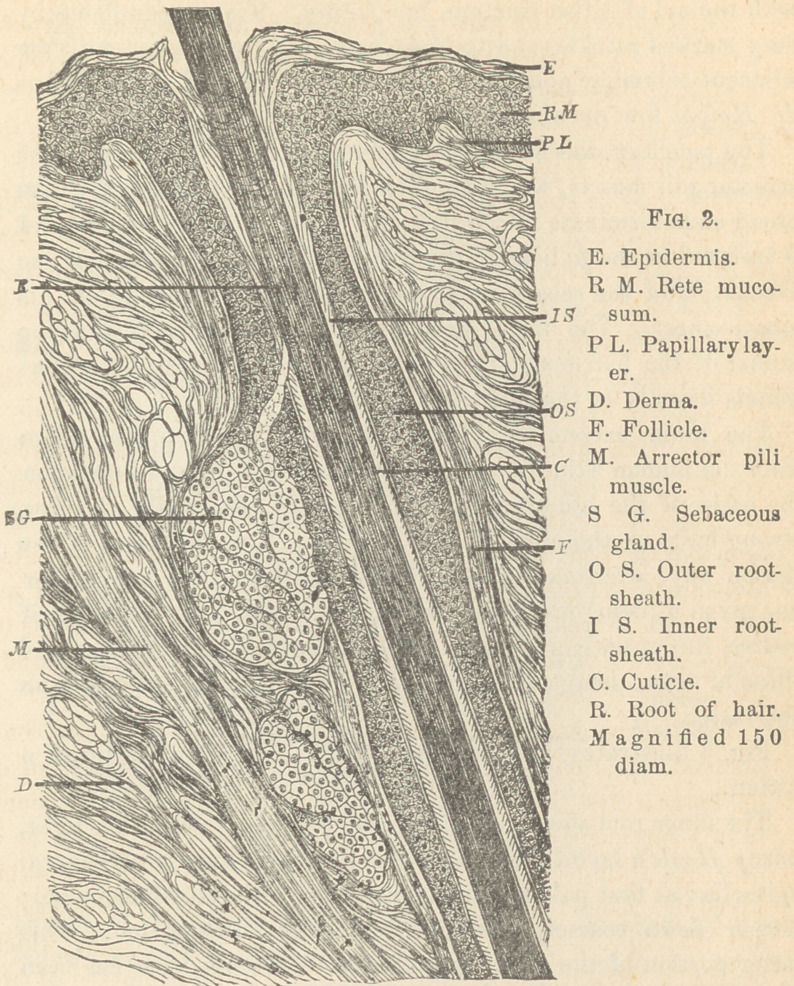


**Fig. 3. f3:**
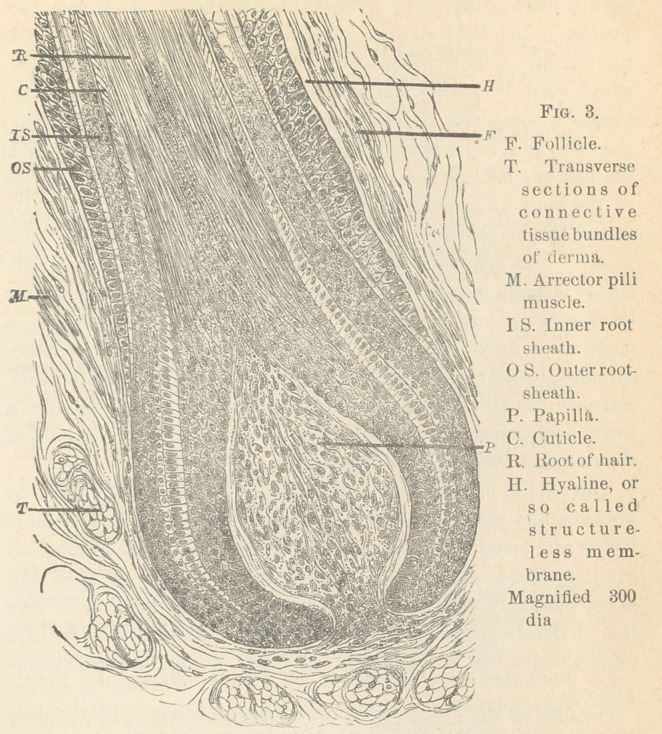


**Fig. 4. f4:**